# Strength, Drying Shrinkage, and Carbonation Characteristic of Amorphous Metallic Fiber-Reinforced Mortar with Artificial Lightweight Aggregate

**DOI:** 10.3390/ma13194451

**Published:** 2020-10-07

**Authors:** Se-Jin Choi, Ji-Hwan Kim, Sung-Ho Bae, Tae-Gue Oh

**Affiliations:** Department of Architectural Engineering, Wonkwang University, 460 Iksan-daero, Iksan 54538, Korea; 3869kjh@naver.com (J.-H.K.); caos1344@naver.com (S.-H.B.); alkjd3@naver.com (T.-G.O.)

**Keywords:** compressive strength, flexural strength, drying shrinkage, amorphous metallic fiber, carbonation, mortar

## Abstract

This paper investigates the strength, drying shrinkage, and carbonation characteristic of amorphous metallic fiber-reinforced mortar with natural and artificial lightweight aggregates. The use of artificial lightweight aggregates has the advantage of reducing the unit weight of the mortar or concrete, but there is a concern that mechanical properties of concrete such as compressive strength and tensile strength may deteriorate due to the porous properties of lightweight aggregates. In order to improve the mechanical properties of lightweight aggregate mortar, we added 0, 10, 20, and 30 kg/m^3^ of amorphous metallic fibers to the samples with lightweight aggregate; the same amount of fiber was applied to the samples with natural aggregate for comparison. According to this investigation, the flow of mortar decreased as the amount of amorphous metallic fiber increased, regardless of the aggregate type. The compressive strength of lightweight aggregate mortar with 10 kg/m^3^ amorphous metallic fiber was similar to that of the LAF0 sample without amorphous metallic fiber after 14 days. In addition, the flexural strength of the samples increased as the amount of amorphous metallic fiber increased. The highest 28-d flexural strength was obtained as approximately 9.28 MPa in the LAF3 sample, which contained 30 kg/m^3^ amorphous metallic fiber. The drying shrinkage of the samples with amorphous metallic fiber was smaller than that of the sample without amorphous metallic fiber.

## 1. Introduction

In general, concrete exerts strong performance against compression, but its tensile strength is very weak—about 8% to 12% of compressive strength—and there is increasing interest in steel-fiber-reinforced concrete to improve such properties as low flexural strength and impact strength [1]. Recently, studies of concrete with thin-shaped amorphous metallic fibers have been actively conducted [2,3,4]. Amorphous metallic fiber has the advantage of reducing CO_2_ and energy consumption in the manufacturing process because this process is simpler than that using normal steel fibers, and there is no subsequent process after the molten iron [4]. In particular, the corrosion or destruction of iron occurs through crystal grain boundaries appearing in the crystalline metal, and the amorphous metal is known to have excellent corrosion resistance and excellent tensile strength [5].

Meanwhile, in order to reduce the weight of ultra-high-rise and large-scale concrete structures, interest in artificial lightweight aggregates having a lighter weight than general aggregates is increasing [6]. The use of artificial lightweight aggregates has the advantage of reducing the unit weight of the cement composite, but there is a concern that mechanical properties such as compressive strength and tensile strength may deteriorate due to the porous properties of lightweight aggregates. Existing studies of cement composites reinforced with amorphous metal fibers have mostly focused on the reduction of the plastic shrinkage of cement composites [7,8] or the improvement of the toughness and impact resistance of high-strength concrete [9,10,11,12,13].

Choi et al. [7] investigated technology for reducing the shrinkage of concrete reinforced with amorphous metallic fibers. According to this study, amorphous metallic fiber is a material developed to improve the shortcomings of steel fibers that has excellent mechanical properties and corrosion resistance. In addition, it can effectively reduce the amount of shrinkage of the mortar and concrete by properly mixing.

Won et al. [8] evaluated the bonding properties between thin amorphous micro-steel fibers and cement composite materials. The bond strength test results with mortar showed that the maximum pull-out load of amorphous micro-steel fiber was larger than that of hooked-type steel fiber.

Lee et al. [10] studied the impact resistance of amorphous-steel-fiber-reinforced cement composites, finding that the composite had a large inhibitory effect on backside destruction by the high-speed impact. It was found that the impact resistance of the cement composites using 30-mm amorphous steel fibers was excellent.

Kim et al. [12] examined the effect that the amorphous metallic fibers have on the attainability of the mixing conditions, the static mechanic properties, and the impact resistance of concrete to those of hooked-end steel fibers. The test results showed that the concrete reinforced with amorphous metallic fibers was more effective at resisting cracking than the concrete reinforced with hooked-end steel fibers.

There are few studies on amorphous-metallic-fiber-reinforced mortar using artificial lightweight aggregates, and it is expected to be helpful in increasing the use of lightweight aggregate concrete if the engineering characteristics of mortar or concrete with artificial lightweight aggregates can be improved using amorphous metallic fibers. In this study, the flow, unit weight, compressive strength, flexural strength, split tensile strength, drying shrinkage, and carbonation characteristic of amorphous-metallic-fiber-reinforced mortar using natural aggregate and artificial lightweight aggregates were investigated in order to improve the mechanical properties of mortar with artificial lightweight aggregates.

## 2. Materials and Methods

### 2.1. Materials

The cementitious materials used in this study were ASTM type I OPC manufactured by the Asia Cement Co. (Seoul, Korea); the blast furnace slag powder was obtained from Daehan Slag Co., Ltd., in Gwangyang, Korea. Natural fine aggregate with a density of 2.60 and a fineness modulus of 2.89 was used. As an artificial lightweight aggregate, the lightweight fine aggregate of KOEN in Jinju, Korea—manufactured by calcining coal ash and dredged soil at about 1100 to 1200 °C—was used.

**Figure 1:**
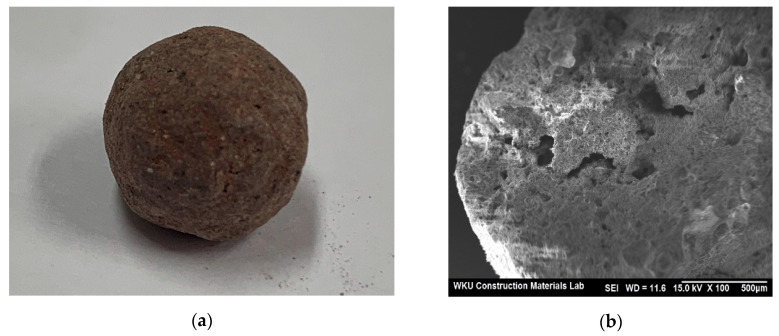
Artificial lightweight aggregate sample: (**a**) Shape, (**b**) SEM image.

Figure 1 shows the shape and scanning electron microscope (SEM) image of the artificial lightweight aggregate, which contains numerous voids.

In general, the surface of the artificial lightweight aggregate first reaches a high temperature in the calcining process, and the surface part is first liquefied and shows a denser shell structure than the inner part [14]. The artificial lightweight aggregate used in this study also showed a similar trend.

**Figure 2:**
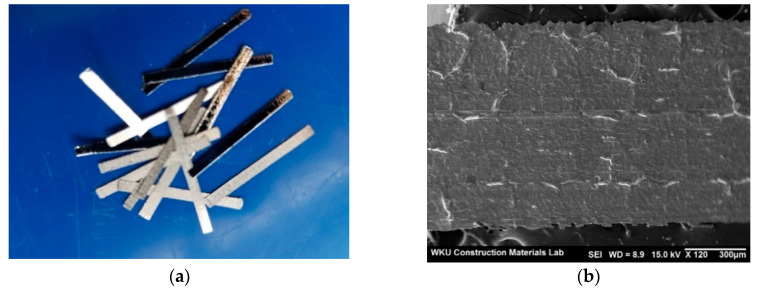
Amorphous metallic fiber: (**a**) Shape, (**b**) SEM image.

Figure 2 shows the shape and SEM image of the amorphous metallic fiber used in this study.

Amorphous metallic fiber with a specific gravity of 7.2 g/cm^3^, a tensile strength of 1400 N/mm^2^, and a length of 15 mm manufactured by SG Group in France was used.

Table 1 and Table 2 show the chemical composition of the cement and BFS (blast furnace slag powder) and the physical properties of the aggregates used.

Table 3 shows the sieve passing ratio of natural and artificial lightweight aggregates used in this study.

### 2.2. Mixing Proportions and Specimen Preparation

In this study, we prepared amorphous-metallic-fiber-reinforced mortar using either natural aggregate or artificial lightweight aggregate. The amount of amorphous metallic fiber used was 0, 10, 20, and 30 kg/m^3^. The water-to-binder ratio was fixed at 0.5. In all mixtures, blast furnace slag powder was used to replace 40% of the weight of cement. The mixing proportions of the mortar mixtures are summarized in Table 4.

The components of the samples were blended in a mechanical mixer, and 50-mm cube molds were prepared for the compressive strength test and unit weight test. Cylindrical molds (∅50 mm × 100 mm) were used for the split-tensile strength test. Bar-type molds (40 mm × 40 mm × 160 mm) were used to measure the flexural strength, drying shrinkage, and accelerated-carbonation test. After 24 h, the strength specimens were removed from their molds and cured at 20 °C in a water tank.

The flow and compressive strength tests of each mix were conducted in accordance with KS L 5105 [15]. The flexural strength and split-tensile strength tests of each mix were conducted in accordance with KS F 2408 [16] and KS F 2423 [17]. The strength test values were the average values of three samples. The unit weights of the samples of each mix were measured in accordance with KS F 2462 [18]. The drying shrinkage test was conducted according to KS F 2424 [19] using a mechanical strain gauge.

**Figure 3:**
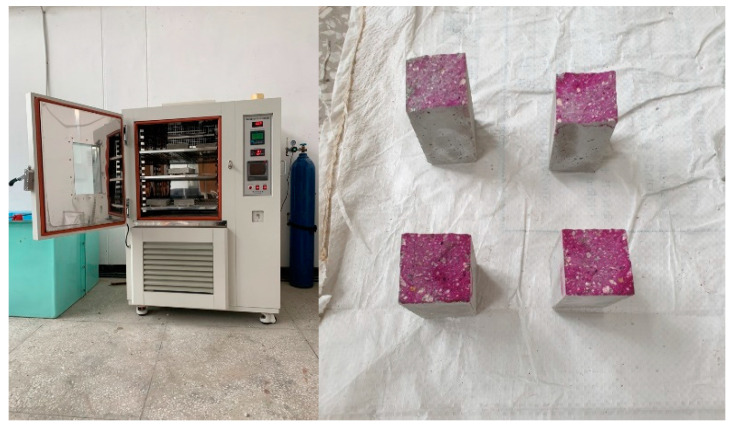
Accelerated-carbonation chamber and samples.

In the carbonation test, the carbonation depth of the specimen was measured using a phenolphthalein solution after the accelerated-carbonation process until the required age in the accelerated-carbonation chamber having a CO_2_ concentration of 5% in accordance with KS F 2584 [20]. Figure 3 shows the carbonation test samples and accelerated-carbonation chamber by CK Corporation of South Korea, Seoul, South Korea, which was used for the carbonation test of the samples.

## 3. Results and Discussion

### 3.1. Mortar Flow

**Figure 4:**
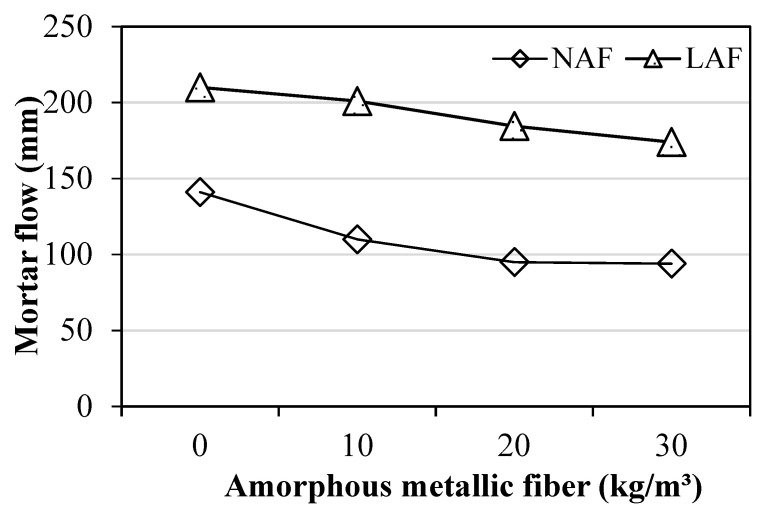
Variation of mortar flow (NAF; Natural aggregate mortar with fiber, LAF; Lightweight aggregate mortar with fiber).

Figure 4 shows the flow value of amorphous-metallic-fiber-reinforced mortar using natural aggregate and artificial lightweight aggregate.

As shown in Figure 4, in both the natural aggregate mixture and artificial lightweight aggregate mixture, the flow of the sample without amorphous metallic fibers was highest. When using natural aggregate, the flow of the samples with amorphous metallic fibers was about 21% to 33% lower than the flow of the NAF0 sample without amorphous metallic fibers. The flow of the sample using artificial lightweight aggregate was larger than that of the sample using natural aggregate, regardless of fiber content. This seems to be due to the round shape of the artificial lightweight aggregate and some of the water absorbed by the artificial lightweight aggregate during the pre-wetting process. In mixtures using artificial lightweight aggregates, the flow also decreased as the amount of amorphous metallic fibers increased. However, the flow reduction of amorphous-metallic-fiber-reinforced mortar using artificial lightweight aggregate was less than that when using natural aggregate.

### 3.2. Unit Weight

**Figure 5:**
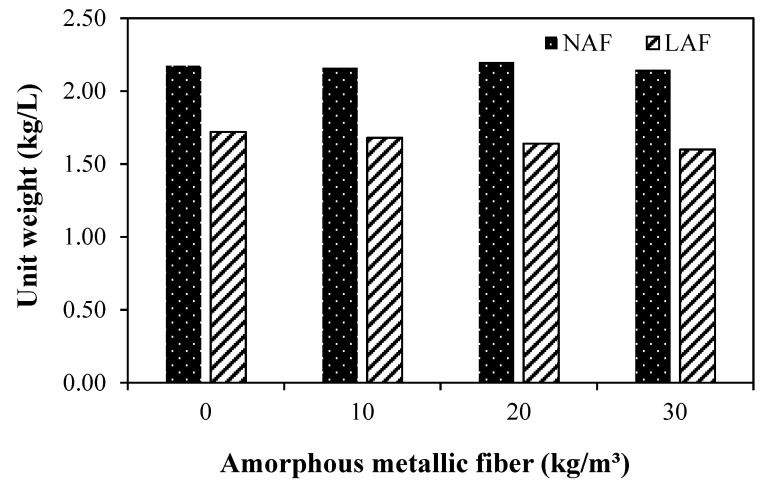
Unit weight.

Figure 5 shows the variation in the unit weight of amorphous-metallic-fiber-reinforced mortar using natural aggregate and artificial lightweight aggregate.

When using natural aggregate, it shows a similar unit weight regardless of the amount of amorphous metallic fiber used. The unit weight of the NAF0 sample without amorphous metallic fiber was about 2.17 kg/L.

The unit weight of the fiber-reinforced natural aggregate mortar sample using 30 kg/m^3^ of amorphous metallic fiber was slightly less than that of the NAF0 sample without fiber. In addition, the unit weight of the mortar samples using artificial lightweight aggregate was 20% or less than that of the mortar samples using natural aggregate. The unit weight of the artificial lightweight aggregate mortar sample without amorphous metallic fibers was about 1.72 kg/L. The unit weight of the sample using amorphous metallic fiber was about 1.60 to 1.68 kg/L, which was slightly less than that of the LAF0 sample without fiber. This seems to be due to the adhesion characteristics and pores in the interface between the amorphous metallic fiber and the mortar matrix.

### 3.3. Compressive Strength

**Figure 6:**
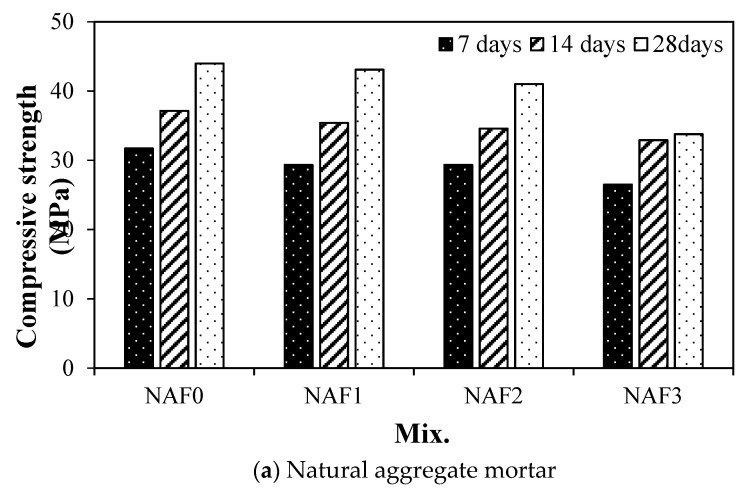

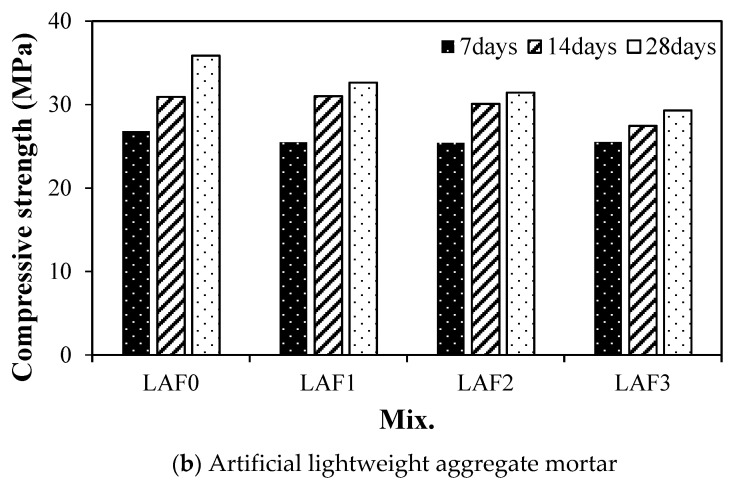
Compressive strength, (**a**) Natural aggregate mortar, (**b**) Artificial lightweight aggregate mortar.

Figure 6 shows the variation in the compressive strength of mortar using natural aggregates according to the amount of amorphous metallic fibers.

As can be seen in the Figure 6a, the 7-day compressive strength of the NAF0 sample without amorphous metallic fibers was about 31.7 MPa, which showed the highest compressive strength of all samples. The compressive strength of both the NAF1 sample and the NAF2 sample, in which 10 kg/m^3^ and 20 kg/m^3^ of amorphous metallic fibers were respectively mixed, was about 29.3 MPa—about 7.5% lower than that of the NAF0 sample. After 14 days, the compressive strength of the NAF0 sample without amorphous metallic fiber was about 37.1 MPa, showing a higher compressive strength than other mixtures, and the samples using amorphous metallic fiber showed a slightly lower compressive strength than the NAF0 sample. After 28 days, the compressive strength of the NAF1 sample with 10 kg/m^3^ amorphous metallic fibers was about 43 MPa, similar to that of the NAF0 sample; the NAF2 sample and NAF3 sample with 20 kg/m^3^ or more amorphous metallic fibers were about 7% to 23% lower than the NAF0 sample without amorphous metallic fibers.

In the case of artificial lightweight aggregate, the compressive strength of the LAF0 sample without amorphous metallic fiber was highest after 7 days, similar to the sample using natural aggregate (Figure 6b). After 14 days, the compressive strengths of the LAF0 sample without amorphous metallic fibers and the LAF1 sample with 10 kg/m^3^ of amorphous metallic fibers were similar, at about 31 MPa. After 28 days, the compressive strength of the LAF0 sample was the highest, at about 35.8 MPa, and the compressive strength of the sample with amorphous metallic fibers decreased as the amount of amorphous metallic fibers increased. When using amorphous metallic fibers in both natural aggregate mortar and artificial lightweight aggregate mortar, the compressive strength of the samples tended to be somewhat reduced or similar to that of the samples without fiber, which is similar to the results of existing literature [1,21] in that the compressive strength enhancement effect of steel-fiber-reinforced concrete is not large.

### 3.4. Flexural Strength

**Figure 7:**
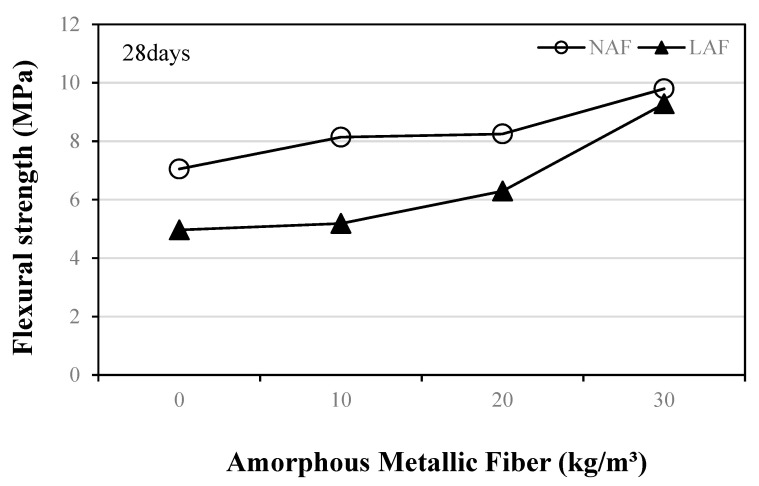
Flexural strength.

Figure 7 shows the variation in flexural strength of amorphous-metallic-fiber-reinforced mortar using natural and artificial lightweight aggregates according to the amount of amorphous metallic fibers after 28 days.

In the case of the mixtures using natural aggregate, the flexural strength of the samples increased as the amount of amorphous metallic fiber increased. The flexural strength of the NAF3 sample using 30 kg/m^3^ of amorphous metallic fiber was about 9.79 MPa, which was about 38.8% higher than that of the NAF0 sample without amorphous metallic fiber. When artificial lightweight aggregate was used, the LAF0 sample without amorphous metallic fiber showed the lowest flexural strength at 4.96 MPa, and the flexural strength of the mixtures increased as the amount of amorphous metallic fiber increased. The flexural strength of the LAF3 sample using 30 kg/m^3^ of amorphous metallic fiber was about 9.28 MPa, which was about 87% higher than that of the LAF0 sample. In addition, the effect of enhancing the flexural strength of the sample due to amorphous metallic fiber reinforcement was higher in the mixtures using artificial lightweight aggregate than in the mixtures using natural aggregate.

**Figure 8:**
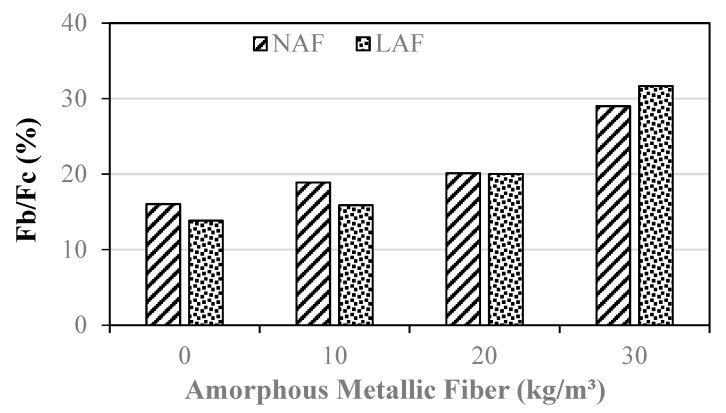
The ratio of flexural strength and compressive strength.

Figure 8 shows the variation of the ratio of flexural strength (Fb) and compressive strength (Fc) of the samples by the amount of amorphous metallic fiber.

In the case of amorphous-metallic-fiber-reinforced mortar using natural aggregate, Fb/Fc was about 18.9% to 29.0%. This value is about 2.9% to 13.0% higher than that of the NAF0 sample without amorphous metallic fibers. In the case of the mixtures using artificial lightweight aggregate, the sample without amorphous metallic fiber showed an Fb/Fc value of about 13.8%, and the Fb/Fc of amorphous-metallic-fiber-reinforced mortar with artificial lightweight aggregate was about 15.9% to 31.7%, which was about 2.1% to 17.9% higher than that of the sample without amorphous metallic fiber. As can be seen in the Figure 8, when the amount of amorphous metallic fiber used was more than 20 kg/m^3^, the Fb/Fc of the artificial lightweight aggregate mortar was similar to or higher than that of the natural aggregate mortar.

### 3.5. Tensile Strength

**Figure 9:**
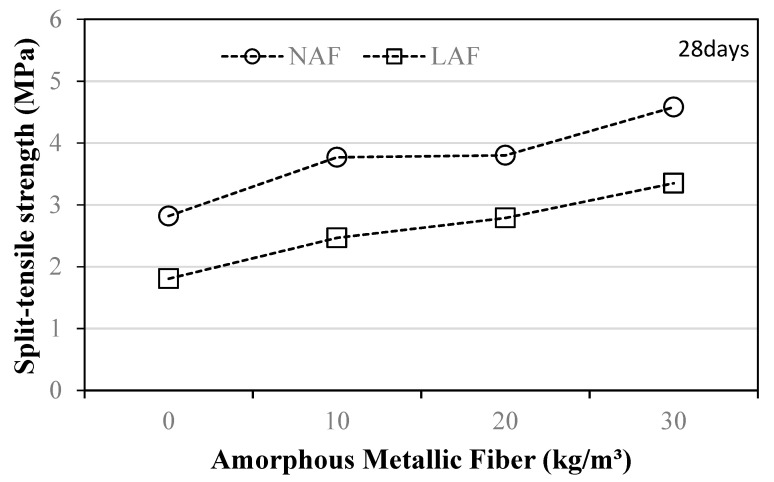
Split-tensile strength.

Figure 9 shows the variation in the split-tensile strength of the mortar sample using natural and artificial lightweight aggregates according to the amount of amorphous metallic fibers after 28 days.

The split-tensile strength of the amorphous-metallic-fiber-reinforced samples increased as the amount of amorphous metallic fibers increased in both natural aggregate mortar and artificial lightweight aggregate mortar. When amorphous metallic fibers were not used, the tensile strength of the natural aggregate mortar was about 2.8 MPa, which was about 55.5% higher than that of the artificial lightweight aggregate mortar. The amorphous-metallic-fiber-reinforced mortar sample using natural aggregate showed a tensile strength of about 3.7 to 4.5 MPa, which was about 32.1% to 60.7% higher than that of the NAF0 sample without amorphous metallic fiber. In addition, the tensile strength of the amorphous-metallic-fiber-reinforced mortar sample using artificial lightweight aggregate was about 2.4 to 3.3 MPa, which was about 33.3% to 83.3% higher than that of the LAF0 sample without amorphous metallic fiber. Similar to the case of flexural strength, the enhancement of the tensile strength of the samples due to the reinforcement of amorphous metallic fibers was greater in the artificial lightweight aggregate mortar than in the natural aggregate mortar.

**Figure 10:**
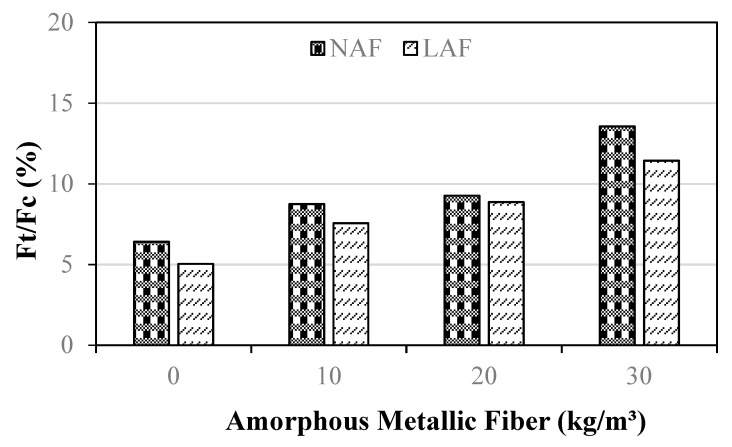
The ratio of tensile strength and compressive strength.

Figure 10 shows the variation in the ratio of the tensile strength (Ft) and compressive strength (Fc) of the samples by the amount of amorphous metallic fiber.

When amorphous metallic fibers were not used, the Ft/Fc values of natural aggregate mortar and artificial lightweight aggregate mortar were 6.4% and 5.0%, respectively. The Ft/Fc values of the amorphous-metallic-fiber-reinforced mortar samples using natural aggregate and artificial lightweight aggregate were 8.7% to 13.5% and 7.5% to 11.4%, respectively. Regardless of aggregate type, the Ft/Fc of the samples increased as the amount of amorphous metallic fiber increased.

### 3.6. Drying Shrinkage

**Figure 11:**
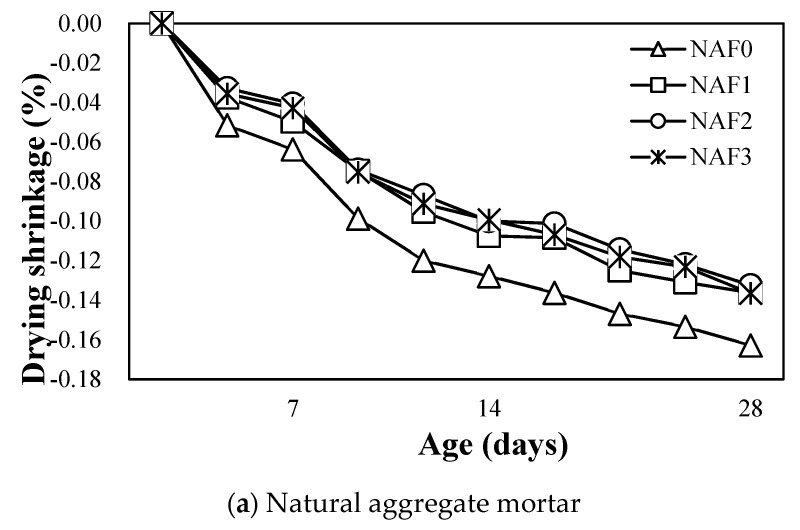

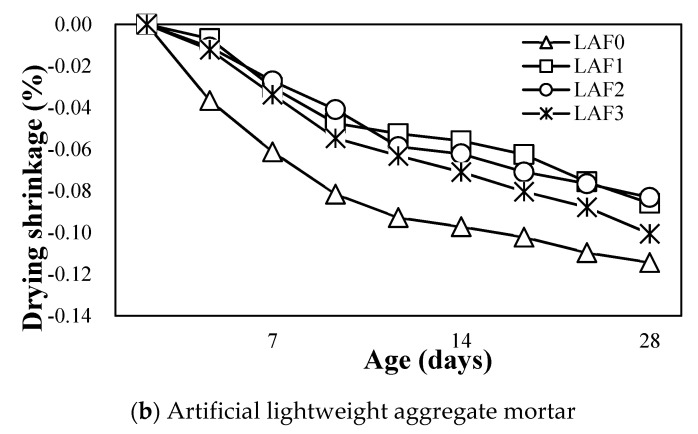
Drying shrinkage, (**a**) Natural aggregate mortar, (**b**) Artificial lightweight aggregate mortar.

Figure 11a shows the variation of drying shrinkage of amorphous-metallic-fiber-reinforced mortar using natural aggregate.

As can be seen in the Figure 11a, the drying shrinkage of the NAF0 sample without amorphous metallic fiber showed the highest value of about 0.163% after 28 days. In the case of amorphous-metallic-fiber-reinforced mortar, regardless of the amount of amorphous fiber used, the drying shrinkage of the samples was about 0.13%—about 20% lower than that of the NAF0 sample.

Figure 11b shows the variation of drying shrinkage of amorphous-metallic-fiber-reinforced mortar using artificial lightweight aggregate. Even in the case of artificial lightweight aggregate mortar, the LAF0 sample without amorphous metallic fibers showed the highest drying shrinkage, and LAF1 and LAF2 samples using 10 and 20 kg/m^3^ of amorphous metallic fibers, respectively, had a drying shrinkage of 0.08% after 28 days—about 27.2% lower than that of the LAF0 sample. Therefore, it is suggested that the drying shrinkage of artificial lightweight aggregate mortar can be effectively reduced by appropriately using amorphous metallic fibers.

### 3.7. Carbonation Depth

**Figure 12:**
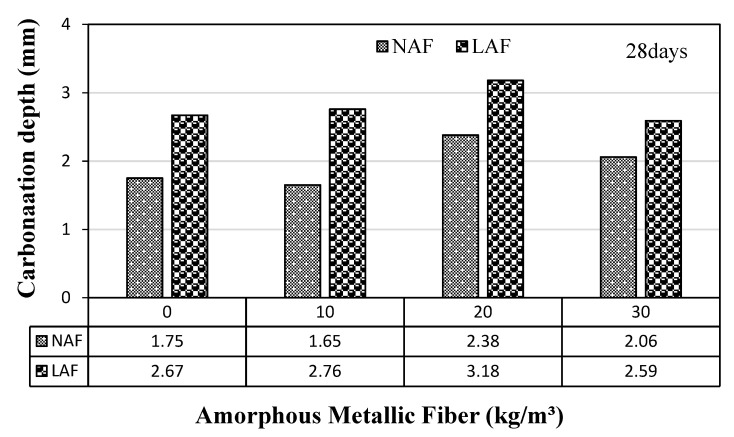
Accelerated-carbonation depth.

The accelerated-carbonation depth of the samples using natural aggregate and artificial lightweight aggregate according to the amount of amorphous metallic fibers is shown in Figure 12.

In the case of natural aggregate mortar, the carbonation depth of the NAF0 sample without amorphous metallic fiber and the NAF1 sample using 10 kg/m^3^ of amorphous metallic fiber was similar. The carbonation depth of the NAF2 sample and NAF3 sample using more than 20 kg/m^3^ of amorphous metallic fiber was somewhat higher than that of the NAF0 sample. Even in the case of artificial lightweight aggregate mortar, the carbonation depth of the sample using amorphous metallic fibers was relatively higher than that of the sample without fiber. When the same amount of amorphous metallic fiber was used, the carbonation depth of artificial lightweight aggregate mortar was higher than that of natural aggregate mortar, which was presumed to be due to the porous characteristics of artificial lightweight aggregate.

## 4. Conclusions

The conclusions of this study can be summarized as follows:

(1) In both the natural aggregate mixture and the artificial lightweight aggregate mixture, the flow of the samples without amorphous metallic fibers was highest. In addition, the flow of the sample using artificial lightweight aggregate was larger than that of the sample using natural aggregate, regardless of fiber content.
(2) The unit weight of the mortar samples using artificial lightweight aggregate was 20% or less than that of the mortar samples using natural aggregate. After 14 days, the compressive strengths of the LAF0 sample without amorphous metallic fibers and the LAF1 sample with 10 kg/m^3^ of amorphous metallic fibers were similar, at about 31 MPa.
(3) The flexural strength of the amorphous-metallic-fiber-reinforced lightweight aggregate mortar sample using 30 kg/m^3^ of amorphous metallic fiber was about 9.28 MPa, which was about 87% higher than that of the LAF0 sample without fibers. In the case of amorphous-metallic-fiber-reinforced mortar using natural aggregates, Fb/Fc was about 18.9% to 29.0%—about 2.9% to 13.0% higher than that of the NAF0 sample without amorphous metallic fibers.
(4) The effect of enhancing the flexural strength and tensile strength of the sample due to amorphous metallic fiber reinforcement was higher in the mixtures using artificial lightweight aggregate than in those using natural aggregate.
(5) The LAF0 sample without amorphous metallic fibers showed the highest drying shrinkage, and the LAF1 and LAF2 samples using 10 and 20 kg/m^3^ of amorphous metallic fibers, respectively, had a drying shrinkage of 0.08% after 28 days, which was about 27.7% lower than that of the LAF0 sample. Therefore, it is suggested that the drying shrinkage of artificial lightweight aggregate mortar can be effectively reduced by appropriately using amorphous metallic fibers.
(6) When the same amount of amorphous metallic fiber was used, the carbonation depth of the artificial lightweight aggregate mortar was somewhat higher than that of the natural aggregate mortar, which was presumed to be due to the porous characteristics of artificial lightweight aggregate.

However, further research is needed to establish the strength development mechanism and respective relationships between the strength properties of cement mortar containing various sizes and volumes of amorphous metallic fibers and water–binder ratio, binder content, durability, etc.

## Figures and Tables

**Table 1:** Chemical composition of cement and blast furnace slag powder (BFS).

Components	SiO_2_	Al_2_O_3_	Fe_2_O_3_	CaO	MgO	K_2_O
Cement	17.43	6.50	3.57	64.40	2.55	1.17
BFS	30.61	13.98	0.32	40.47	6.43	0.60

**Table 2:** Physical properties of aggregates.

Type of Sand	Fineness Modulus	Surface Dry Density (g/cm^2^)	Oven Dry Density (g/cm^2^)	Water Absorption Ratio (%)	Unit Weight (kg/L)
Natural sand (NS)	2.89	2.60	-	1.00	1427
Lightweight sand (LS)	4.61	1.77	1.63	8.71	1010

**Table 3:** Sieve passing ratio of aggregates.

Type of Sand	Sieve Passing Ratio (%)						
	10 mm	5 mm	2.5 mm	1.2 mm	0.6 mm	0.3 mm	0.15 mm
Standard	100	100	100	85	60	30	10
	100	95	80	50	25	10	2
NS	100	100	93.75	73.50	52	20	6
LS	100	99.75	38.50	1.25	0.25	0.20	0.20

**Table 4:** Mix proportions of mortar.

Mix.	W/(C+BFS)	Water (kg/m^3^)	Cement (kg/m^3^)	BFS (kg/m^3^)	Natural Sand (kg/m^3^)	Lightweight Sand (kg/m^3^)	Fiber (kg/m^3^)
NAF0	0.5	170	204	136	735	-	0
NAF1	0.5	170	204	136	735	-	10
NAF2	0.5	170	204	136	735	-	20
NAF3	0.5	170	204	136	735	-	30
LAF0	0.5	170	204	136	-	502	0
LAF1	0.5	170	204	136	-	502	10
LAF2	0.5	170	204	136	-	502	20
LAF3	0.5	170	204	136	-	502	30
